# Clopidogrel-Induced Recurrent Polyarthritis

**DOI:** 10.1177/2324709613500239

**Published:** 2013-08-05

**Authors:** Sahil Agrawal, Joseph Harburger, Gary Stallings, Nikhil Agrawal, Jalaj Garg

**Affiliations:** 1 Department of Internal Medicine, Westchester Medical Center, New York Medical College, Valhalla, NY, USA; 2 Division of Cardiology, Department of Internal Medicine, Westchester Medical Center, New York Medical College, Valhalla, NY, USA

**Keywords:** clopidogrel, plavix, arthritis, arthralgia

## Abstract

Clopidogrel is an oral thienopyridine and together with aspirin is a component of dual antiplatelet therapy for the prevention of stent thrombosis after intracoronary stent placement. The common adverse effects from its use are an increased risk of bleeding, neutropenia, and rash. Arthralgia and backache are also known to occur with its use. There have been case reports linking arthritis with the use of clopidogrel. We describe the case of a 64-year-old man who reported symptoms of fever and joint pains following initiation of therapy with clopidogrel. Acute-phase reactants were elevated. Laboratory and radiologic testing were unremarkable. Incidentally, he reported experiencing a similar arthritis after he received a loading dose of clopidogrel prior to a diagnostic coronary angiography in the past. The symptoms improved dramatically on discontinuation of clopidogrel. There was no recurrence of symptoms with prasugrel. This describes possibly the second incidence of recurrent arthritis with clopidogrel therapy.

## Introduction

Platelets have a pivotal role in atherothrombosis and are critical participants in acute thrombotic complications. Antiplatelet therapy has therefore been the cornerstone of pharmacologic management of coronary artery disease. The thienopyridines are a group of pro-drugs whose active metabolite irreversibly inhibits the ADP binding P2Y_12_ receptor on the platelet membrane surface.^1,2^ This ADP–P2Y_12_ interaction normally plays a crucial role in the amplification of platelet aggregation and subsequent clot genesis.^3^ Clopidogrel is a commonly prescribed thienopyridine. The efficacy and safety of clopidogrel has been established in several large, randomized, controlled trials. The Clopidogrel versus Aspirin in Patients at Risk of Ischemic Events (CAPRIE) trial demonstrated that long-term administration of clopidogrel to patients with atherosclerotic vascular disease was more effective than aspirin in reducing the combined risk of ischemic stroke, myocardial infarction, or vascular death with a similar safety profile.^4^ The Clopidogrel in Unstable Angina to Prevent Recurrent Ischemic Events (CURE) trial showed a sustained, incremental benefit when clopidogrel was added to aspirin in patients with unstable angina and non-Q-wave myocardial infarction.^5^ Current percutaneous coronary intervention guidelines recommend dual antiplatelet therapy with aspirin and clopidogrel for at least 1 month after implantation of bare metal stents and at least 12 months after implantation of drug-eluting stents.^6^ Early discontinuation of dual antiplatelet therapy has been identified as a risk factor for stent thrombosis in patients with drug-eluting stents.^7^ Clopidogrel has also been used as an alternative to aspirin in patients who have hypersensitivity related to its use.^8^ The most common side effects from clopidogrel use are gastrointestinal problems and an increased risk of bleeding, especially when used in combination with aspirin;^9^ with back pain and arthralgia each occurring in 6% of patients.^10^ We report a unique case of recurrent migratory arthritis that we hypothesize was induced by reexposure to clopidogrel.

## Case Report

A 64-year-old man underwent stenting of the left circumflex coronary artery using a drug-eluting stent for worsening anginal symptoms 2 weeks prior to his presentation to us. He reported fever that started 4 days after the procedure and right shoulder pain and neck stiffness for 1 day. Clopidogrel was added at the time of the procedure per standard guidelines. The fever did not resolve despite an interim course of antibiotics. On examination he had a temperature of 101.3°F, pulse of 98 beats per minute, blood pressure of 110/70 mm Hg and respiratory rate of 12/minute. There was markedly limited passive and active range of motion at the right shoulder joint and cervical spine. Examination of the cardiovascular, respiratory, gastrointestinal, genitourinary, and nervous systems was unremarkable. A lumbar puncture was performed and blood and urine cultures were sent. Antibiotics were started awaiting culture results. The next day he developed painful swelling and erythema of the bilateral wrists with restricted range of motion, and the fever persisted. His past medical history included hypertension and medically managed coronary artery disease. His home medications included aspirin, atorvastatin, and metoprolol in addition to clopidogrel. He denied having a rash, mucosal ulcers, diarrhea, dysuria, cough, tick bites, recent travel, drug allergies, or family history of arthritis. He did, however, report receiving a single loading dose of clopidogrel with a similar self-resolving migratory arthritis at the time of a prior coronary angiography the previous year. The patient recalled that the arthritis had occurred around a week after the clopidogrel dose and that he had a fever but no rash. At that time, percutaneous coronary intervention was not performed and clopidogrel was not continued. White cell count and differential, electrolytes, and kidney and liver function were within normal limits. Serum uric acid concentration (4.4 mg/dL) was normal. Radiographs of the shoulder and wrists demonstrated only degenerative changes. Sterile synovial fluid with few white cells was aspirated from the shoulder joint. Erythrocyte sedimentation rate (ESR; 89 mm/h) and C-reactive protein (CRP; 15 mg/dL) were elevated. Serum Lyme ELISA and Brucella serologies were negative. Parvovirus IgG but not IgM was detected reflecting past exposure. Tests to detect antinuclear antibodies, antibodies to extractable nuclear antigens, antineutrophil cytoplasmic antibodies, rheumatoid factor, anticyclic citrullinated antibodies, and the HLA B27 allele were negative. Serum protein electrophoresis and immunofixation were unremarkable. Genetic testing for familial Mediterranean fever was also negative. The proximity of clopidogrel exposure to symptom onset led us to postulate that the patient’s arthritis was clopidogrel induced. This suspicion was further heightened by prior occurrence of these symptoms under similar circumstances. Clopidogrel was discontinued in favor of prasugrel. Colchicine and indomethacin were used to treat the arthritis. Antibiotics were discontinued when cultures returned negative. Within the next 2 days, symptoms of joint inflammation had much improved with good range of motion at the previously affected joints. After 1 month of follow-up there has been no recurrence of symptoms.

## Discussion

Our patient had acute migratory polyarthritis of indeterminate etiology. Alternative diagnoses considered were autoimmune arthritis, infectious arthritis, reactive arthritis, gout, pseudogout, serum sickness, and familial Mediterranean fever. Physical exam, laboratory testing, and radiographic imaging revealed no clear cause of our patient’s symptoms. Arthritis and serum sickness are classified as type B (unpredictable hypersensitivity) adverse drug reactions. There are reports of acute arthritis in relation to maintenance dose clopidogrel therapy^10-13^; in addition to a single report of similar symptoms with only a loading dose.^14^ In one instance the same phenomena were reinduced by readministration of the drug.^15^ Cheema et al studied 62 patients with clopidogrel hypersensitivity. Two patients (3%) reported symptoms of fever and arthralgia with a median time to development of clopidogrel hypersensitivity of approximately 5 days.^16^ The case that we describe reports a similar chronology of symptom onset and distribution of afflicted joints as described in prior cases. Given the timing of symptoms in relation to the initiation of clopidogrel and occurrence of a similar arthritis following a previous clopidogrel exposure, we suspect our patient developed acute arthritis secondary to clopidogrel hypersensitivity. Clopidogrel-related arthritis may therefore be an uncommon manifestation of clopidogrel hypersensitivity. Both prasugrel^17,18^ and ticlopidine^19^ have been used as safe alternatives following this phenomenon without recurrence of arthritis. We report the third case in which clopidogrel was replaced with prasugrel with resolution of symptoms and the second case of recurrence of clopidogrel-induced arthritis.

## Conclusion

We recommend considering clopidogrel as a cause of acute arthritis in patients receiving this drug and avoid using clopidogrel in patients with a prior unexplained episode of arthritis temporally related to clopidogrel therapy.

## Clopidogrel-Induced Arthropathy: A Review of the Literature

We searched PubMed using various combinations of the keywords “Clopidogrel,” “Plavix,” “arthritis,” and “arthralgia.” The search resulted in 29 results, which were scrutinized by 2 independent reviewers. Fourteen results of interest were further reviewed independently. These included 10 case reports of arthritis associated with the use of clopidogrel. We had access to full text for 7 of these 10 cases. A note was made in relation to the following characteristics in each case: age, sex, presence of rash, pruritus, fever, time to onset of symptoms following initiation of clopidogrel, affected joints, and levels of inflammatory markers (ESR, CRP). Table 1 summarizes the case reports. All but one case subject was male. No age predilection was observed in our review. The most common accompanying symptoms were fever, rash, and pruritus, typical of a drug-mediated idiosyncratic reaction. The rash was described as maculopapular in all cases in which a rash was present. Onset of symptoms occurred 2 to 3 weeks after maintenance clopidogrel was initiated, except in 1 case in which symptoms occurred after 3 days. This chronology suggests a possible immunologic basis for these symptoms. The only reported case of arthritis following a single loading dose without maintenance therapy occurred 48 hours after exposure.^14^ The arthritis was migratory and symmetric when it involved multiple joints. Some cases had mono or oligo-articular involvement. Both small and large joints were affected. Synovitis was the hallmark finding on examination while X-ray imaging did not reveal any specific findings. All cases had an associated elevation of acute inflammatory markers to varying degrees: ESR values ranged between 47 and 86 mm/h, CRP values where available were between 21 and 408 mg/L. None of the cases had eosinophilia, while an elevated level of serum IgE was reported in 1 case.^15^ Autoantibodies were absent except for 1 case with low positive levels of antinuclear antibodies.^11^ The same case also had a positive IgG for parvovirus. Improvement was reported in all cases shortly after clopidogrel was discontinued. Patients additionally received steroids or nonsteroidal anti-inflammatory drugs as described in Table 1. Our patient had fever but no rash or pruritus. There was an inflammatory arthritis of the right shoulder and bilateral wrist and hand joints that occurred in a migratory pattern, approximately 2 weeks after clopidogrel maintenance therapy was initiated. Interestingly, our patient reported similar symptoms approximately 1 year earlier following a loading dose of clopidogrel. No additional doses of clopidogrel were given at the time of this prior episode and symptoms resolved. To our knowledge, there has been only 1 documented case of recurrent clopidogrel-induced arthritis.^15^ This case occurred following deliberate readministration of clopidogrel.

**Table 1.** Demographic and Clinical Characteristics of Patients With Clopidogrel-Induced Arthropathy.

Study	Age (years)	Sex	Symptoms	Onset Following Administration	Joints Involved	Inflammatory Markers	Treatment (Including Discontinuing Clopidogrel)
Coulter and Montandon^17^	52	Male	F, P	2 weeks	Knees, hips, shoulders, hands, elbows	ESR 68, CRP 171	Prasugrel + steroids
Kawashiri et al^15^	54	Male	F, P	3 weeks	Shoulder, hips, hands, knees, ankles	ESR 81, CRP 81	
Kanadiya et al^18^	50	Male	F, R	3 days	Shoulders, right MCP	CRP 21	Ticlopidine + prednisone
Tayyareci et al^14^	72	Male	F	48 hours	Right knee	ESR 76, CRP 140	Indomethacin
Blauwet and Matteson^11^	72	Male	R	3 weeks	Wrists, knees	ESR 54	Prednisone
Garg et al^12^	76	Female	P	2 weeks	MCP	ESR 86, CRP 81	
Garg et al^12^	63	Male	—	3 weeks	Right knee	ESR 47	

Abbreviations: F, fever; P, pruritus; R, rash; ESR, erythrocyte sedimentation rate; CRP, C-reactive protein.

The basis of this drug reaction is uncertain. Garcia et al provided compelling evidence for the pro-inflammatory activity of clopidogrel in an animal model.^20^ In their experiment, rats treated with clopidogrel had increased joint diameter, elevated plasma levels of pro-inflammatory cytokines, an elevated neutrophil count, and an increased platelet count compared to untreated rats. Platelet factor 4 levels were unaffected by clopidogrel treatment, suggesting that the inflammatory effect of clopidogrel may be independent of its effect on platelets. The authors advised careful use of clopidogrel in patients with history of arthritis. Ticlopidine, another thienopyridine, has also been reported to cause similar symptoms, suggesting a possible class effect.^21^ We recommend considering clopidogrel as a cause of acute arthritis in patients receiving this medication. Resolution of symptoms on substituting clopidogrel with an acceptable alternative would strongly suggest clopidogrel-associated arthritis.
